# iSPOT: A Web Tool for the Analysis and Recognition of Protein Domain Specificity

**DOI:** 10.1002/cfg.104

**Published:** 2001-10

**Authors:** Barbara Brannetti, Andreas Zanzoni, Luisa Montecchi-Palazzi, Gianni Cesareni, Manuela Helmer-Citterich

**Affiliations:** 1 Department of Biology University of Rome Tor Vergata Rome Italy; 2 Department of Biology University of Rome Tor Vergata Via della Ricerca Scientifica Rome 00133 Italy

## Abstract

Methods that aim at predicting interaction partners are very likely to play an important role in the interpretation of genomic information. iSPOT (iSpecificity Prediction Of Target) is a web tool (accessible at http://cbm.bio.uniroma2.it/iSPOT) developed for the prediction of protein-protein interaction mediated by families of peptide recognition modules. iSPOT accesses a database of position specific residue-residue interaction frequencies for members of the SH3 and PDZ protein domain families. The software utilises this database to provide a score for any potential domain peptide interaction.

iSPOT: 1. evaluates the likelihood of the interaction between any of the peptides contained in an input protein and a list of domains of the two different families; 2. searches in the SWISS-PROT database for potential partners of a query domain; and 3. has access to a repository of all the domain/target peptide interaction data.


**Comparative and Functional Genomics**
*Comp Funct Genom* 2001; **2**: 314–318.
**DOI:** 10.1002/cfg.104

**Conference Review**

### iSPOT: a web tool for the analysis and recognition of protein domain specificity

A presentation for the ESF workshop 'Proteomics: Focus on Protein Interactions'

Barbara Brannetti, Andreas Zanzoni, Luisa Montecchi-Palazzi, Gianni Cesareni and Manuela Helmer-Citterich*
*Department of Biology, University of Rome Tor Vergata, Rome, Italy*

**Correspondence to:*
*M. Helmer-Citterich, Department of Biology, University of Rome Tor Vergata, Via della Ricerca Scientifica, 00133 Rome, Italy.*
*E-mail: citterich@uniroma2.it*

Received: 28 June 2001
Accepted: 27 July 2001

### Abstract

**Methods that aim at predicting interaction partners are very likely to play an important role in the interpretation of genomic information. iSPOT (iSpecificity Prediction Of Target) is a web tool (accessible at http://cbm.bio.uniroma2.it/iSPOT) developed for the prediction of protein-protein interaction mediated by families of peptide recognition modules. iSPOT accesses a database of position specific residue-residue interaction frequencies for members of the SH3 and PDZ protein domain families. The software utilises this database to provide a score for any potential domain peptide interaction.**

**iSPOT: 1. evaluates the likelihood of the interaction between any of the peptides contained in an input protein and a list of domains of the two different families; 2. searches in the SWISS-PROT database for potential partners of a query domain; and 3. has access to a repository of all the domain/target peptide interaction data. Copyright © 2001 John Wiley & Sons, Ltd.**

**Keywords: prediction of specificity; molecular recognition; protein domains; computational methods; web tools; homology modelling**

### Introduction

The formation of protein complexes is often mediated by families of protein modules that are found repeatedly in the proteome and that have evolved to recognize specific protein surface features. The ability to infer the binding specificity of any given domain from limited experimental data would represent an important tool to extend our understanding of the protein network inside the cell.

Different methods have been used to predict the binding partners of a given domain when a list of binding peptides has been determined experimentally. These include profile search methods [2,3] and pattern matching methods [5,6]. More sophisticated methods based on homology modelling and energy minimisation techniques can be applied when the three-dimensional structure of the domain in complex with at least one ligand peptide is known (for a review, see [7]).

The SPOT procedure [1] was developed to provide a software tool that would elaborate on the experimental data obtained by screening peptide repertoires in order to infer the recognition specificity of any element of a protein module family. The application of SPOT, in contrast to profile and pattern matching methods, is not restricted to domains for which experimental binding data is available. Furthermore (unlike techniques based on molecular dynamics) SPOT does not require that the domain three-dimensional structure is available and provides a prediction as long as the sequence of the domain member under study can be confidently aligned to a domain of known structure from the same family.

In essence, SPOT is based on the assembly of a 20 by 20 matrix for each pair of domain peptide residues that are deduced to interact, even loosely, from the inspection of the available three-dimensional structure of peptide-domain complexes (Figure 1). The 20 by 20 matrices contain information

Copyright © 2001 John Wiley & Sons, Ltd.

on the frequency with which amino acid x (on the peptide ligand) is observed whenever the contacting amino acid y is present in the receptor domain. It is then assumed that the interaction between a domain/peptide pair can be approximated by the sum of independent interactions between their contacting residues and the "domain specific matrices" are used to estimate, position after position, the likelihood that two proteins would interact. In this approximation, the binding preference of domains of unknown specificity can be inferred when there is at least some sequence identity in the binding regions with the domains whose experimental data have been utilised to fill the 'domain specific matrices'. In this case, an evaluation of the unknown binding *consensi* can be obtained from all the interaction data derived from other similar domains of the same family. The reliability of the prediction depends on the level of sequence identity, in the region involved in target recognition, between the query domain and the domains whose experimentally determined binding data have been used to train the software.

The procedure was developed for application to the SH3 domain family [1]. In principle, however, it can be extended to any protein interaction domain family for which at least one structure of a domain/ligand complex is known, in order to permit the identification of the residues that make specific contacts. Furthermore, the domain family and the ligand peptides should be sufficiently homogeneous to permit their confident alignment in the binding region. Finally, and most importantly, experimental data on the preferred ligands of a number of members of the domain family must be available.

### Results and discussion

At the workshop in Villa Mondragone we reported the development of the iSPOT interface in order to make the SPOT procedure accessible to Internet

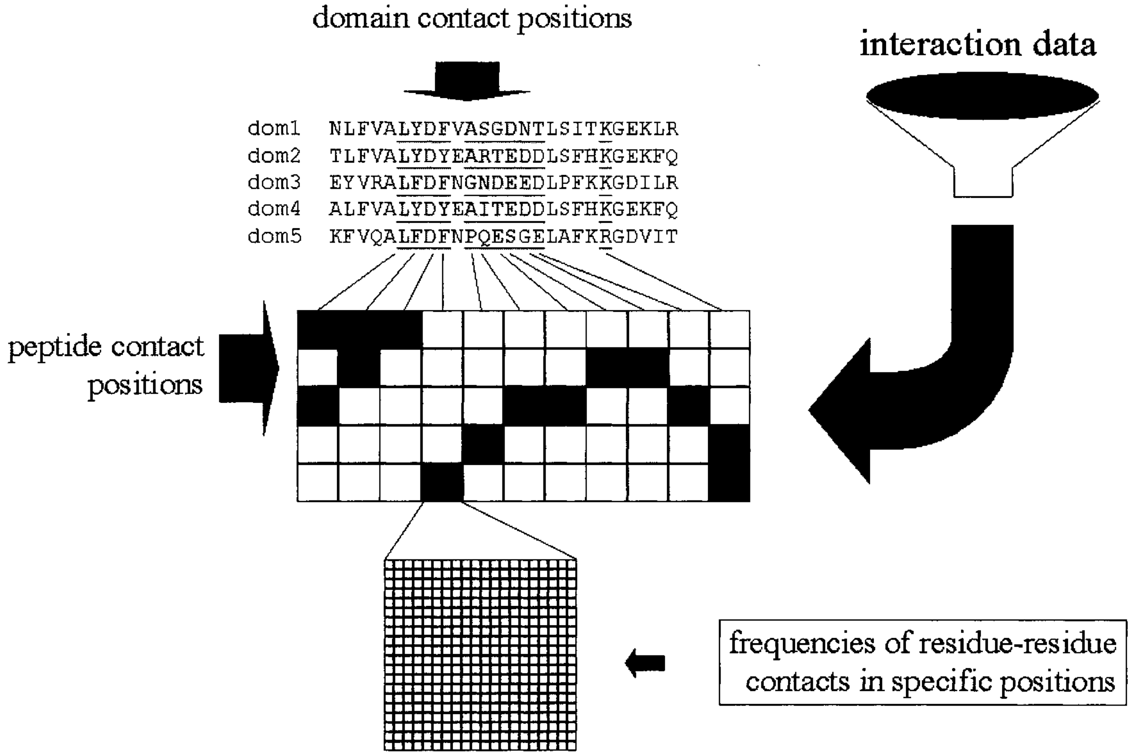

**Figure 1.** Domain-specific matrix.
The positions that make contact in the domain-peptide complex are identified from the three dimensional structures available in the PDB: the columns in the matrix refer to the residues in the domain that are involved in peptide recognition, the rows describe the ligand contacting positions. Each domain/peptide contact pair (a black matrix element in the figure) is described by a 20 by 20 matrix containing the frequencies of the contacting residues in the experimentally identified domain/peptide pairs. The *form* of the matrix is derived from structural data, while its *content* is calculated from the available experimental interaction data

Copyright © 2001 John Wiley & Sons, Ltd.

users. The results can be obtained in html format and as text files via e-mail.

The iSPOT procedure has also been applied to PDZ domains [8] and to MHC class I molecules (Montecchi-Palazzi *et al.*, manuscript in preparation). Both applications are made available through the iSPOT home page (Figure 2, [4]).

iSPOT permits the user to ask the following questions:

(i) Which SH3 or PDZ domains are likely to bind to any given protein/peptide sequence (Figure 3a)?
(ii) Which protein (or peptide), contained in the SWISS-PROT database, is a potential ligand of a query SH3 or PDZ domain (Figure 3b)?
(iii) Which experimental binding data are available, if any, for the protein domains of interest?

From the iSPOT home page (Figure 2), one first selects a domain family to access the list of available iSPOT features.

### A protein sequence versus one or more SH3/PDZ domains

A protein sequence in FASTA format can be entered and the SPOT program launched to rank a

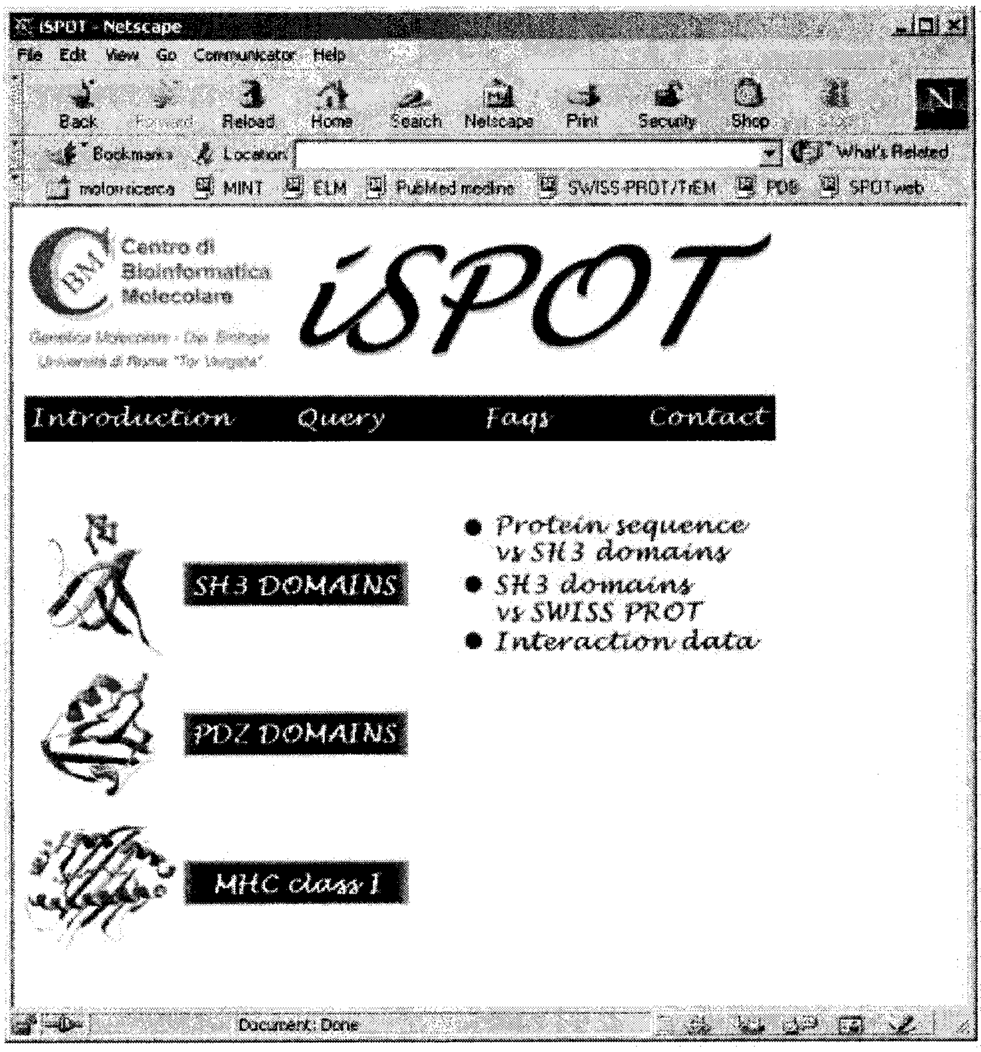

**Figure 2.** iSPOT home page.
The user can click on any of the domains that have been already implemented in the SPOT procedure in order to access the different iSPOT features

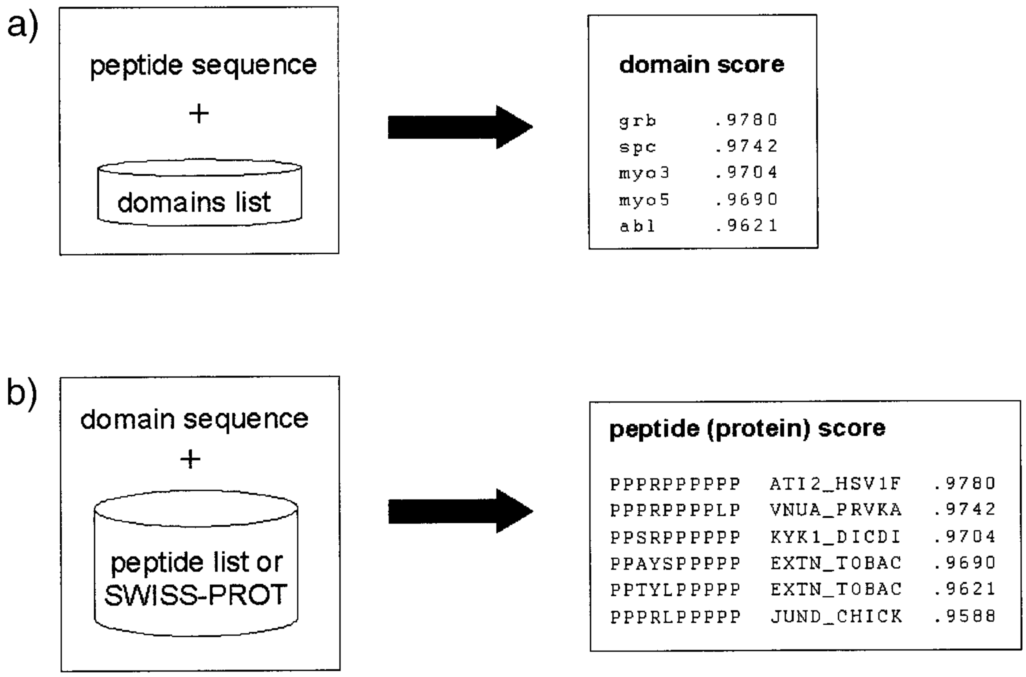

**Figure 3.** iSPOT-predicted results.
a) The procedure can rank different domains of a given family according to the probability of binding a query input peptide.
b) SPOT can be used to search peptide lists or protein databases (such as SWISS-PROT) for proteins potentially interacting with a query domain selected subset of SH3 domains according to the probability that they would bind any decapeptide in the query protein. Three different precompiled lists of SH3 domains can be selected: only mammals, only yeast or both. Furthermore, the user can select one or more domains from the whole list of SH3 domains present in the SPOT multiple alignment [1]. If PDZ domains are analysed, a list of query carboxy-terminal peptides can be entered and their predicted interaction probability with a list of available PDZ domains can be obtained [8]. The results of the SPOT procedure are immediately displayed and also sent by e-mail to the user.

### One SH3 or PDZ domain versus the SWISS-PROT database

The SPOT procedure is very fast and can therefore be applied to genomic data. The peptides in the SWISS-PROT database that are ranked high when queried with any SH3 or PDZ domain present in the SPOT multiple alignment can easily be retrieved. The SPOT alignment is manually maintained, as previously described [1]. Users interested in predictions of ligands of SH3 or PDZ domains that are not yet present in the SPOT alignment can request that we add their domain of interest.

### Interaction data

The complete list of domains in the SPOT multiple alignment is reported. A subset of the domains contribute to the domain-specific matrix with interaction data. Interaction data in the form of peptide lists can be viewed, together with the appropriate references. Users can submit lists of binding peptides for a domain of their interest *via* a submission form, together with a reference. These data will be used to enrich the domain-specific matrix and added to the iSPOT database of interaction data.

### Future developments

We also report a new feature, which will soon be available on the iSPOT page. Users will have access to the database of frequencies of position-specific residues that can be interpreted to obtain suggestions about mutations that should change the

Copyright © 2001 John Wiley & Sons, Ltd.

binding specificity of a query domain. In other words, SPOT will suggest which residues of the domain should be mutated into which other residues in order to specifically lower or increase its affinity for peptides of defined sequence.

### Acknowledgements

This work was supported by EEC project QLRI-CT-2000-00127 and by the CNR target project in Biotechnology.

### References

1. Brannetti B, Via A, Cestra G, Cesareni G, Helmer-Citterich M. 2000. SH3-SPOT: an algorithm to predict preferred ligands of different members of the SH3 gene family. *J Mol Biol* **298**: 313–328.
2. Gribskov M, Mclachlan AD, Eisenberg D. 1987. Profile analysis: detection of distantly related proteins. *Proc Natl Acad Sci U S A* **84**: 4355–4358.
3. Gribskov M, Veretnik S. 1996. Identification of sequence pattern with profile analysis. *Methods Enzymol* **266**: 198–212.
4. iSPOT: **http://cbm.bio.uniroma2.it/iSPOT**
5. Parker KC, Bednarek MA, Coligan JE. 1994. Scheme for ranking potential HLA-A2 binding peptides based on independent binding of individual peptide side-chains. *J Immunol* **152**: 163–175.
6. Rammensee HG, Bachmann J, Emmerich NPN, Bachor AO, Stevanovic S. 1999. SYFPEITHI: database for MHC ligands and peptides motifs. *Immunogenetics* **50**: 213–219.
7. Sternberg MJE. Protein structure prediction – Principles and approaches. In *Protein Structure Prediction, a Practical Approach,* Sternberg MJE (ed.), IRL Press at Oxford University Press; 1–30.
8. Vaccaro P, Brannetti B, Montecchi-Palazzi L, *et al.,* (in press). Distinct binding specificity of the multiple PDZ domains of the human INADL, a human protein with homology to INAD from *Drosophila melanogaster*. *J Biol Chem.*

Copyright © 2001 John Wiley & Sons, Ltd. 
